# Army Order—Relative Rank of Army and Navy Officers

**Published:** 1850-10

**Authors:** 


ARMY ORDER—RELATIVE RANK OF ARMY AND NAVY OFFICERS.

*Washington, Sept. 26th,* 1850.

The following Army Order was issued by the War Department on the 21st inst.:

"The House of Representatives having on the 18th of July, 1850, adopted a resolution, requesting the President to communicate to that Honorable body his views of the rules and regulations which should be established by law on certain subjects therein mentioned relating to rank in the army and navy, the President directs that a board of officers of the army be assembled, who shall deliberate on so much of said resolution as appertains to the army, and shall consult with any similar board, composed of officers of the navy, that may be appointed for the purpose, in relation to so much of said resolution as relates to the relative rank of the army and navy."

The following officers of the army are appointed members of the board: Gen. Scott, president; Gen. Jessup, Gen. Wool, Col. Crane, Col. Waite, Surgeon Mower, Paymaster Hunter, Lt. Col. Scott, recorder. The board will assemble at Washington, on Monday, the 14th of October, and after closing their proceedings, will report to the War Department their views and opinions on the subjects submitted to them.

We hope that the subject of *assimilated rank* will be brought before this board, and that our medical brethren in both services will at last have justice done them.

